# Escalated Oxycodone Self-Administration Is Associated with Activation of Specific Gene Networks in the Rat Dorsal Striatum

**DOI:** 10.3390/ijms26157356

**Published:** 2025-07-30

**Authors:** Ammanuel Y. Wabreha, Michael T. McCoy, Jean Lud Cadet, Atul P. Daiwile

**Affiliations:** Molecular Neuropsychiatry Research Branch, DHHS/NIH/NIDA Intramural Research Program, 251 Bayview Boulevard, Baltimore, MD 21224, USA; ammanuel.wabreha@nih.gov (A.Y.W.); mmccoy@intra.nida.nih.gov (M.T.M.); jcadet@intra.nida.nih.gov (J.L.C.)

**Keywords:** oxycodone, potassium channels, RNA sequencing, dorsal striatum, self-administration

## Abstract

The diagnosis of opioid use disorder (OUD) is prevalent due to increased prescribing of opioids. Long-term oxycodone self-administration can lead to addiction-like behavioral responses in rats. Herein, we sought to identify molecular pathways consequent to long-term exposure to oxycodone self-administration. Towards that end, we used male Sprague Dawley rats that self-administered oxycodone for 20 days according to short-(ShA, 3 h) and long-access (LgA, 9 h) paradigms. LgA rats escalated their oxycodone intake and developed into 2 phenotypes, labeled Long-access High (LgA-H) and Long-access Low (LgA-L) rats, based on their escalation. RNA sequencing analysis revealed the LgA-H has significantly different DEGs in comparison to other groups. DAVID analysis revealed the participation of LgA-H DEGs in potassium transport. RT-PCR analysis of striatal samples validated the increased levels of potassium channels. Since these increases correlated with oxycodone intake, we believe potassium channels are potential targets for the treatment of oxycodone use disorder

## 1. Introduction

The opioid epidemic continues to pose a significant challenge to public health [1,2], despite some efforts to curb excessive prescription of opioid drugs like oxycodone that are often used for pain management [3,4]. In the USA, for example, overdose deaths involving prescription opioids significantly increased from 3442 in 1999 to 14,716 in 2022 [5]. Biochemically, oxycodone is a semisynthetic opioid drug with a relatively long history of being prescribed throughout the world [6,7]. Its administration by patients leads to tolerance and dependence to oxycodone [8,9], followed by eventual switching to and misuse of stronger opioids including heroin with subsequent development of opioid use disorder (OUD). This psychiatric disorder is associated with neuropsychiatric and neuropathological complications [10,11,12] and, regrettably, with overdose-related deaths [13,14]. These clinical and pathological sequelae draw attention to an urgent need for the development of more effective anti-OUD treatments. Pharmacological approaches to OUD have targeted opioid receptor-related systems [15,16,17,18]. These approaches are often associated with various levels of complications including misuse and diversion [19]. Although intravenous self-administration (IVSA) models have paved the way for mechanistic investigations, a better understanding of the neurobiological consequences of repeated oxycodone use in humans is necessary.

Towards that end, animal models that mimic aspects of OUD, including oxycodone self-administration (SA), have been used to investigate potential biochemical and molecular pathways affected by this drug [11,20,21]. Previous studies utilizing intravenous self-administration (IVSA) of oxycodone in rodents have employed both short-access (ShA; 1–3 h/day) and long-access (LgA; 6+ h/day) models, with LgA models consistently demonstrating a variation in escalation of intake over time [22,23,24]. Methodological variations across laboratories include differences in training protocols, cue exposure, reinforcement schedules, and inclusion of punishment paradigms to assess compulsivity [22,25,26]. More recently, intermittent access schedules have emerged as a tool to model human-like patterns of episodic opioid intake [27].

As a first step towards identifying global oxycodone-induced molecular alterations in the brain, we have also used the drug SA model to measure global changes in gene expression in the dorsal striatum, a brain region pivotal to habitual drug-taking behaviors [28,29,30,31], and some of the clinical manifestations of substance use disorders [32,33]. Among the molecular pathways likely to contribute to oxycodone-induced neuroadaptations, potassium channels are thought to play an important role because of their potential roles in the development and maintenance of substance use disorders (SUDs) [34]. These channels control the flow of potassium ions across the neuronal membrane, allowing them to influence action potential firing, neurotransmitter release, and neuronal responsiveness to stimulants [34,35,36,37]. Potassium channels are also involved in the fine-tuning of neural circuits within the dorsal striatum, which is implicated in reward and reinforcement [38]. Altered potassium channel activity in this brain region can influence the balance between goal-directed and habitual behaviors, potentially contributing to the maladaptive plasticity observed in OUD [39,40]. Understanding how these channels are regulated during drug exposure offers valuable insights into the molecular basis of OUD. Herein, using RNA sequencing, we studied the global transcription changes in the dorsal striata of oxycodone self-administered rats and the potential roles of potassium channels in OUD.

## 2. Results

### 2.1. Rats Exposed to LgA Oxycodone Self-Administration Escalate Their Drug Intake over Time

**Figure 1:**
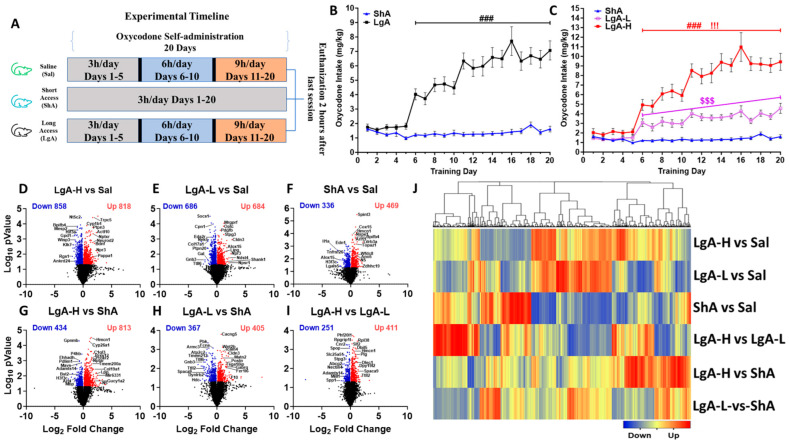
Insights into oxycodone self-administration: Experimental timeline, behavioral data, RNA sequencing analysis (**A**) Experimental timeline, Saline (*n* = 8), Short-access (ShA) (*n* = 10) rats, and Long-access (LgA) (*n* = 18). (**B**) Oxycodone intake by LgA and ShA groups. (**C**) LgA rats show two distinct intake phenotypes, high (LgA-H) (*n* = 11) and low (LgA-L) (*n* = 7), based on their drug intake. Volcano plots (**D**) LgA-L vs. Sal, (**E**) LgA-L vs. Sal, (**F**) ShA vs. Sal, (**G**) LgA-H vs. ShA, (**H**) LgA-L vs. ShA, (**I**) LgA-H vs. LgA-L illustrating the number of significant genes (*p*  <  0.05) between each pairwise comparison. (**J**) Hierarchical clustering for DEGs that met the criterion of 1.5-fold change and *p*  <  0.05, with blue indicating downregulated genes, and red indicating upregulated genes. Key to statistics: ### = *p* < 0.001, comparison of LgA-H to ShA rats; !!! *p* < 0.001, comparison of LgA-H to LgA-L rats; $$$ *p* < 0.001, comparison of LgA-L to ShA rats.

Figure 1A shows the experimental timeline. As described previously [20], rats self-administered either saline or oxycodone (0.1 mg/kg/infusion) under an FR1 schedule for 20 days. We analyzed the behavioral data using repeated measures two-way ANOVA with groups (ShA vs. LgA) and training days as factors. We observed significant effects for group (F_(1, 49)_ = 37.6, *p* < 0.0001), oxycodone intake (F_(4.467, 218.9)_ = 13.91, *p* < 0.0001), and group × oxycodone intake interaction (F_(19, 931)_ = 12.37, *p* = 0.001). Post-hoc test showed LgA rats had greater oxycodone intake than ShA rats (Figure 1B).

As noted for oxycodone [41] and methamphetamine [42] previously, LgA rats pressed the lever differentially to access oxycodone. We thus performed a second-degree polynomial regression analysis to compare oxycodone acquisition and rate of change of oxycodone intake over time, for individual rats, as reported in previous papers [41,42,43,44]. We found that some LgA rats significantly increased their oxycodone intake over the 20-day period, whereas others did not escalate their intake. We identified rats that escalated their oxycodone intake by looking for an increase in their tolerance levels over time [45]. Animals that escalated their oxycodone intake over 20 days of SA were called Long-access High (LgA-H), whereas those that did not escalate were named Long-access Low (LgA-L). Subsequently, we reanalyzed the behavioral data with three phenotypes LgA-H, LgA-L, and ShA. Two-way ANOVA showed significant effects for groups (F_(2, 48)_ = 86.30, *p* < 0.0001), oxycodone intake (F_(5.491, 263.5)_ = 33.42, *p* < 0.0001), and group × oxycodone intake interaction (F_(38, 912)_ = 14.1, *p* = 0.001). Post-hoc analysis revealed that LgA-H self-administered more oxycodone than LgA-L and ShA, but such a difference was not seen when LgA-L was compared to ShA (Figure 1C), with ShA rats failing to escalate their intake also.

We therefore reasoned that the behavioral differences in terms of oxycodone intake might be due to different drug-induced molecular neuroadaptations in the three oxycodone SA phenotypes. In order to test this idea, we performed RNA sequencing to identify potential global transcriptional changes in the dorsal striata of these rats. The dorsal striatum is an important structure that is involved in the neuroanatomical circuit that subsumes addictive processes because it mediates the transition from goal-directed drug use to habitual drug-seeking behavior [46]. It integrates dopaminergic inputs associated with the brain’s reward circuitry, supporting motor planning and action selection [47,48,49]. With repeated drug exposure, neural adaptations in the dorsal striatum are thought to underlie the shift toward automatic, stimulus-driven responding, commonly seen in OUD [39,40,50]. Given its role in these behavioral changes, examining the dorsal striatum can provide valuable insight into the mechanisms driving this behavior.

### 2.2. RNA Sequencing Identifies Specific Differentially Expressed Genes in the Dorsal Striatum of LgA-H, LgA-L, and ShA Rats

**Figure 2:**
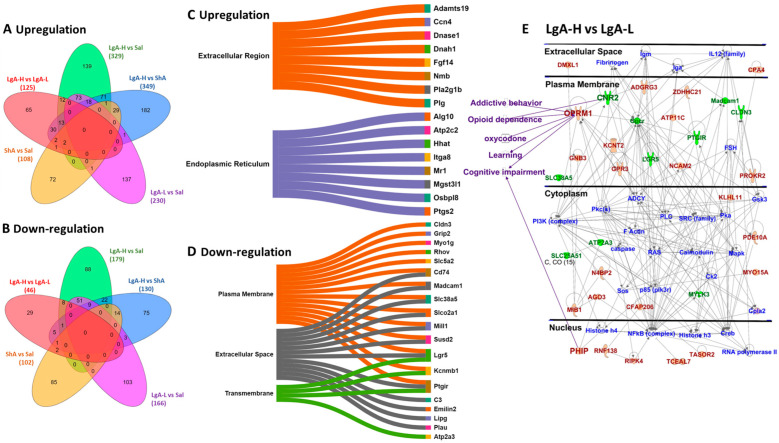
Identification of gene and molecular network in the LgA-H animals in comparison to LgA-L. (**A**) The Venn diagram shows significant upregulated genes (*p*  <  0.05, fold change 1.5 F). (**B**) This Venn diagram shows significant downregulated genes (*p*  <  0.05, fold change 1.5 F). Sankey diagrams (Sankeymatic.com/build) reveal molecular functions for genes that were (**C**) upregulated and (**D**) downregulated in the LgA-H vs. LgA-L comparison. (**E**) Ingenuity Pathway Analysis (IPA, https://digitalinsights.qiagen.com/products-overview/discovery-insights-portfolio/analysis-and-visualization/qiagen-ipa/, accessed on 26 July 2025) shows the molecular networks for DEGs in the LgA-H vs. LgA-L comparison. The red color represents upregulated genes, whereas the green color represents downregulated genes, and the blue color represents interacting partners.

We used RNA sequencing to identify transcriptional changes in the striatum, which might be associated with differences in oxycodone intake. The results of these comparisons are shown as volcano plots (Figure 1D–I). Analysis of RNA sequencing data using log_2_ fold changes and log_10_ *p* values revealed 1676 differentially expressed genes (DEGs) in LgA-H vs. CT, 1370 DEGs in LgA-L vs. CT, 805 DEGs in ShA vs. CT, 1247 DEGs in LgA-H vs. ShA, 772 DEGs in LgA-L vs. ShA, and 662 DEGs in LgA-H vs. LgA-L (Figure 1D–I). We set a more restrictive cut-off of equal to or greater than 1.5-fold (*p* = 0.05) and identified 1300 DEGs in six pairwise comparisons. These are illustrated in a hierarchical clustering heat-map (Figure 1J). Venn diagrams illustrated in Figure 2A,B show unique and shared up- and downregulated DEGs identified in the pairwise comparisons.

### 2.3. Striatal Genes Are Differentially Expressed in LgA-H

In the present study, the LgA-H rats showed significantly higher oxycodone intake than intake by LgA-L and ShA rats (Figure 1C). We therefore reasoned that DEGs identified in LgA-H rats might be related, in part, to oxycodone use disorder in humans. We performed pairwise comparisons to identify DEGs that were uniquely expressed in LgA-H rats in comparison to Sal, LgA-L, and ShA rats (Figure 2A,B). Database for Annotation, Visualization, and Integrated Discovery (DAVID) analysis was performed to gain more insight into possible biological functions that these DEGs might participate in. These biological functions were shown as Sankey plots (Figure 2C,D). The Qiagen Ingenuity Pathway Analysis (IPA) software (v01-23-01) was also used to identify gene networks that might be associated with higher oxycodone intake. Figure 2E identified involvement of several unique DEGs in addictive behavior, opioid dependence, oxycodone use, learning and cognitive impairment.

**Figure 3:**
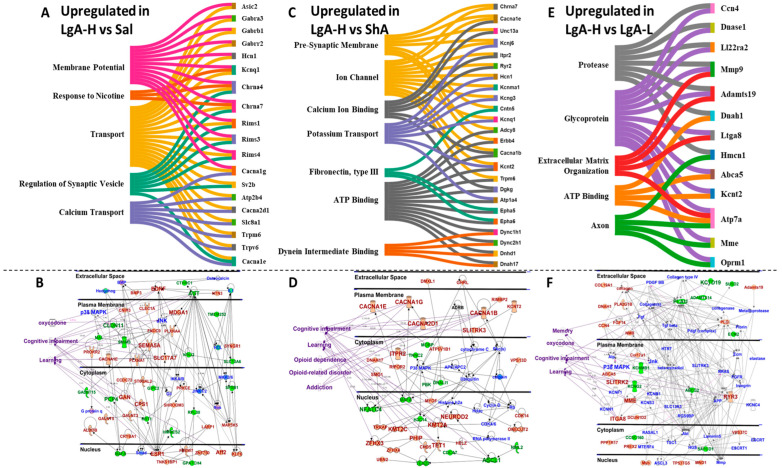
Identification of altered molecular networks in the LgA-H rats in comparison to Sal, ShA, and LgA-L. (**A**) Sankey diagrams revealed molecular functions of genes that were upregulated. (**B**) represents IPA gene networks in the LgA-H vs. Sal comparison. (**C**) Sankey diagrams revealed molecular functions of genes that were upregulated. (**D**) IPA illustrates gene networks in the LgA-H vs. ShA comparison. (**E**) Sankey diagrams revealed molecular function of genes that were upregulated. (**F**) IPA shows gene networks in the LgA-H vs. LgA-L comparison. The red color represents upregulated genes, whereas the green color represents downregulated genes, and the blue color illustrates interacting partners.

We also identified biological functions and gene networks for all DEGs found in LgA-H rats in comparison to Sal (Figure 3A,B), ShA (Figure 3C,D), and LgA-L rats (Figure 3E,F). These DEGs include genes involved in synaptic membrane potential, calcium transport, ion channel, potassium channels, ATP binding, and axon (Figure 3A,C,E). IPA analysis also identified DEGs with involvement in the manifestations of oxycodone use, opioid dependence and related disorders, addiction, cognitive impairments, and learning/memory (Figure 3B,D,F).

### 2.4. Increased Oxycodone Intake Is Associated with Upregulation of Potassium Channel Genes in the Dorsal Striatum of LgA-H Rats

**Figure 4:**
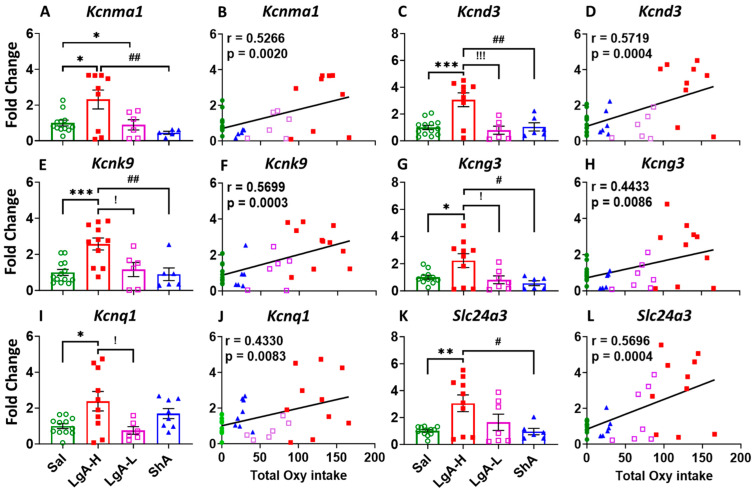
Potassium channels showed increased expression in the dorsal striatum of LgA-H rats. (**A**) Kcnma1, (**C**) Kcnd3, (**E**) Kcnk9, (**G**) Kcng3, (**I**) Kcnq1, and (**L**) Slc24a3. This increased expression of potassium channels was found to positively correlate with oxycodone intake (**B**) Kcnma1, (**D**) Kcnd3, (**F**) Kcnk9, (**H**) Kcng3, (**J**) Kcnq1, and (**K**) Slc24a3. Key to statistics: *, **, *** = *p* < 0.05, 0.01, 0.001, comparison LgA-H, LgA-L, or ShA to saline rats; #, ## = *p* < 0.05, 0.01, comparing LgA-H to ShA rats; !, !!! = *p* < 0.05, 0.001 comparing LgA-H to LgA-L rats.

As stated above, behavioral differences in the patterns of oxycodone intake are thought to be associated with different molecular neuroadaptations. IPA and Sankey diagrams have identified potassium channels as a cluster of genes that might be involved in the behavioral manifestation of LgA-H and LgA-L. These included *Kcnma1*, *Kcnk9*, *Kcnq1*, *Kcnd3*, *Kcng3,* and *Slc24a3* mRNAs that were upregulated in the RNA sequencing data. Potassium channels are known to play critical roles in membrane potential, action potential, neurotransmitter release, and rhythmic firing of neurons [51,52,53], thereby regulating the synaptic plasticity [54,55], cognitive function [56,57,58], and behavior [59]; therefore, we chose to validate the RNA sequencing data by quantitative PCR (Figure 4).

Figure 4 shows that LgA-H rats displayed significant higher mRNA levels for *Kcnma1* [F_(3, 28)_ = 5.323, *p* = 0.0050] (Figure 4A), *Kcnd3* [F_(3, 30)_ = 10.30, *p* < 0.0001] (Figure 4C), *Kcnk9* [F_(3, 32)_ = 8.090, *p* = 0.0004] (Figure 4E), and *Kcng3* [F_(3, 30)_ = 4.773, *p* = 0.0078] (Figure 4G) when compared to LgA-L, ShA, and Sal. We also observed significant positive linear correlation between mRNA levels for *Kcnma1* (r = 0.5266, *p* = 0.0020), *Kcnd3* (r = 0.5719, *p* = 0.0004), *Kcnk9* (r = 0.5699, *p* = 0.0003), and *Kcng3* (r = 0.4433, *p* = 0.0086) with total oxycodone intake at the end of 20 days of self-administration (Figure 4B,D,F,H). In addition, the mRNA level of *Kcnq1* was significantly upregulated [F_(3, 32)_ = 4.450, *p* = 0.0101] in the LgA-H phenotype when compared to LgA-L and Sal (Figure 4I), with a significant positive linear relationship to total oxycodone intake (r = 0.4330, *p* = 0.0083) (Figure 4J). Moreover, we found that Slc24a3 expression significantly increased [F_(3, 31)_ = 5.397, *p* = 0.0042] also in the LgA-H rats when compared to ShA and Sal (Figure 4K), with a significant positive correlation to total oxycodone intake (r = 0.5696, *p* = 0.0004) (Figure 4L).

### 2.5. LgA-L and ShA Rats Showed Differential Gene Expression in the Dorsal Striatum

LgA-L rats were given similar access to oxycodone as the LgA-H rats. Yet, those rats self-administered less oxycodone (Figure 1C). We thus performed comparative analyses between LgA-L vs. Sal, LgA-L vs. ShA, and LgA-L vs. LgA-H to identify genes that were specifically changed in LgA-L rats (Supplementary Figure S1A,B). Additionally, we conducted the following comparisons: ShA vs. Sal, ShA vs. LgA-L, and ShA vs. LgA-H, in order to identify genes that were changed in the ShA rats (Supplementary Figure S1C,D) that were exposed to less oxycodone, took less of the drug, and did not escalate their intake. DAVID annotation and Sankey diagrams revealed functional classification of DEGs in LgA-L or ShA rats in comparison to Sal (Supplementary Figures S2 and S3). IPA analysis showed that the DEGs in LgA-L or ShA rats were also involved in oxycodone use, opioid dependence and related disorders, addiction, cognitive impairments, and learning/memory (Supplementary Figures S2 and S3), suggesting that exposure to any amount of oxycodone might be detrimental.

**Figure 5:**
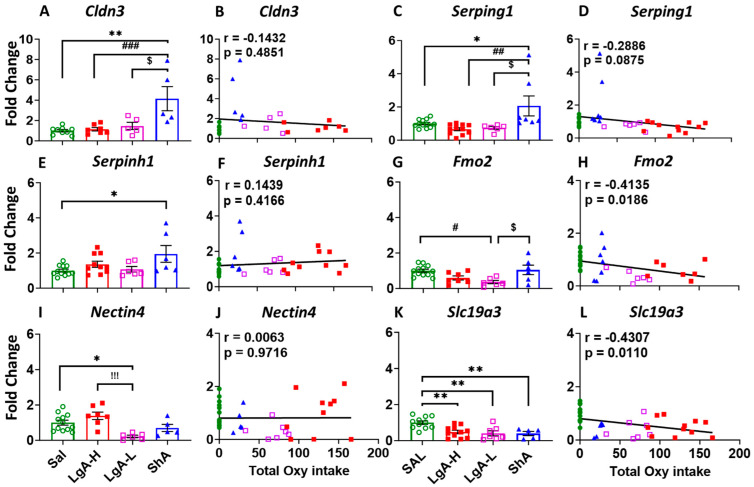
Real-time quantitative PCR of DEGs of other genes. (**A**) Cldn3, (**C**) Serping1, and (**E**) Serpinh1, showed changed expression, which was not correlated with oxycodone intake, illustrated in (**B**) Cldn3, (**D**) Serping1, and (**F**) Serpinh1 in ShA rats. (**G**) Fmo2, and (**I**) Nectin4 showed decreased expression in the dorsal striatum of LgA-L rats, with (**H**) Fmo2 but not (**J**) Nectin4 showing negative correlation with oxycodone intake. (**K**,**L**) Expression of Slc19a3 was decreased in all oxycodone-exposed rats, without any correlation with oxycodone intake. Key to statistics: *, ** = *p* < 0.05, 0.01, comparison of LgA-H, LgA-L, or ShA to saline rats; #, ##, ### = *p* < 0.05, 0.01, 0.001 comparing LgA-H rats to ShA rats; $ = *p* < 0.05, comparison of LgA-L rats to ShA rats; !!! = *p* < 0.001, comparison of LgA-H rats to LgA-L rats.

In order to validate the changes in these animals, we used quantitative PCR to measure the expression of some of these genes. ShA rats showed significant increase in the expression of Claudin 3 (*Cldn3*) [F_(3, 22)_ = 8.193, *p* = 0.0008] and serpin family G member 1 (*Serping1*) [F_(3, 32)_ = 5.969, *p* = 0.0024] compared to Sal, LgA-L, and LgA-H, with no correlation with total oxycodone intake (Figure 5A–D). Serpin family H member 1 (*Serpinh1*) was found to be significantly upregulated [F_(3, 30)_ = 3.513, *p* = 0.0270] in the ShA rats compared to Sal (Figure 5E), with no significant correlation with oxycodone intake (Figure 5F).

Expression of flavin-containing monooxygenase 2 (*Fmo2*) was found to be significantly down regulated [F_(3, 27)_ = 5.183, *p* = 0.0059] in the LgA-L rats when compared to ShA and Sal (Figure 5G), with a significant negative linear relationship to oxycodone intake (r = −0.4135, *p* = 0.0186) (Figure 5H). Moreover, *Nectin4* [F_(3, 26)_ = 7.617, *p* = 0.0008] was also found to be decreased in LgA-L rats in comparison to Sal and LgA-H (Figure 5I), with no correlation to oxycodone intake (Figure 5J). Interestingly, expression of solute carrier family 19 member 3 (*Slc19a3)* was down regulated [F_(3, 30)_ = 7.526, *p* = 0.007] in LgA-H, LgA-L and ShA compared to Sal (Figure 5K), with significant negative correlations with oxycodone intake (r = −0.4307, *p* = 0.0110) (Figure 5L).

## 3. Discussion

The present study assessed behavioral responses in rats exposed to different schedules of oxycodone self-administration. We then used RNA sequencing to measure global mRNA expression in the dorsal striata of oxycodone-exposed rats. We found that some rats exposed to a long-access oxycodone schedule took a large amount of the drug (LgA-H) in contrast to some other rats (LgA-L). Rats exposed to a short-access schedule took less oxycodone and did not escalate their intake. RNA sequencing data identified molecular pathways associated with different behavioral patterns of oxycodone intake. Interestingly, the levels of several potassium channels: potassium calcium-activated channel subfamily M alpha 1 (*Kcnma1*), potassium two pore domain channel subfamily K member 9 (*Kcnk9*), potassium voltage-gated channel subfamily Q member 1 (*Kcnq1*), potassium voltage-gated channel subfamily D member 3 (*Kcnd3*), potassium voltage-gated channel modifier subfamily G member 3 (*Kcng3*) and solute carrier family 24 member 3 (*Slc24a3*) mRNAs were increased in the dorsal striatum of LgA-H rats in comparison to other rats.

As previously reported for both methamphetamine (METH) [42,43,44], cocaine [45], and oxycodone [11,20,41], rats given long access to drugs were split into two groups, with some animals increasing their drug intake but not others. These behavioral differences might be related to their sensitivity to the specific drug. Sensitivity to opioids appears to be related, in part, to control of dopamine release [60]. Clinical studies have documented that some humans feel the effects of drugs as more reinforcing [61,62,63,64,65], with increased sensitivity to drugs putting individuals at greater risk of developing addictive disorders [66,67,68,69,70].

### 3.1. Involvement of Potassium Channel Genes in High Oxycodone Intake

Our RNA sequencing and PCR data identified potassium channel mRNAs as relevant to the high oxycodone intake. Through our analysis we identified specific potassium channels to be of interest due to their large fold changes in the experiments after exposure to oxycodone (see above) and their potential roles in the dorsal striatum and the brain in general [34]. Potassium channels work in cells to influence the shape and duration of action potentials by modulating membrane potential [71]. Of specific interest, *Kcnma1* is a calcium-gated potassium channel that plays a role in the brain’s neuronal excitability and synaptic plasticity [72]. *Kcnk9* is a gene that encodes a protein called TASK3, a potassium channel important in memory formation [73]. In addition, *Kcnd3*, *Kcng3,* and *Kcnq1* are voltage-gated potassium channels that influence firing patterns in the brain [74,75]. Moreover, *Slc24a3* is a sodium/potassium/calcium exchanger that plays a role in maintaining both Ca and K homeostasis [73]. In addition, evidence reviewed by McCoy et al. (2021) [34] also implicates the dysregulation of potassium channels in neurological disorders, including METH use disorder [76], autism [77], and epilepsy [71,78]. Thereby, it is fair to reason that the potassium channels we identified may play an important role in synaptic plasticity [54,55], cognitive function [56,57,58], and behavioral responses [59] associated with the symptoms of high oxycodone use.

Of specific relevance to our discussion about the role of potassium in the observed behavioral responses to oxycodone is the report that drugs that target potassium channels might be promising in the treatment of pain [79] since oxycodone is a known opioid anti-analgesic [6,7]. Altogether, these observations suggest that more experiments are needed to evaluate the role of potassium channels in SUDs.

### 3.2. Molecular Mechanisms Associated with Low Oxycodone Intake

In the present study, LgA-L rats showed decreased expression of flavin-containing dimethylaniline monooxygenase 2 (*Fmo2*) and *Nectin4*. The changes in *Fmo2* levels are of interest because *Fmo2* is known to be involved in the metabolism of nicotine [80,81] and has been implicated in SUDs [82,83,84,85]. Some studies have documented alterations in the levels of *Fmo2* mRNA in response to methamphetamine [82,83], amphetamine [84], and heroin [85]. Nectine4, a cell adhesion molecule, was also reported to be involved in OUD [86,87] and appeared to be an indicator of continued opioid use [86]. Since plasma *Nectin4* levels have also been reported to be increased in methadone [86] and ketamine abusers [88], it might be of interest to test the idea that the plasma *Nectin4* levels might be related to the therapeutic effects of these two drugs.

## 4. Materials and Methods

### 4.1. Animals

Male Sprague Dawley rats that weighed (350–400 g, 12–15 weeks old) were procured from Charles River (Boston, MA, USA). The rats were housed in a controlled setting with free access to food and water. This environment operated under a reversed 12 h light/dark cycle (lights off 09:30–21:30) [41]. All self-administration sessions began (~9:00 AM every day) at the start of the dark phase of the light/dark cycle. All experimental procedures adhered to the guidelines outlined in the National Institutes of Health (NIH) Guide for the Care and Use of Laboratory Animals and were approved by the NIDA (National Institute of Drug Abuse) Animal Care and Use Committee at the Intramural Research Program (IRP), protocol number 18-MNPB-11.

### 4.2. Intravenous Surgery

Rats were first anesthetized with ketamine (50 mg/kg) and xylazine (5 mg/kg). One end of the polyurethane catheter was surgically inserted into the right jugular vein, while the other end was mounted to the rats’ back [20]. Post-surgery, rats received intraperitoneal injections of buprenorphine (0.1 mg/kg) for pain relief. Rats were allowed to recover for 7 days before the start of self-administration training.

### 4.3. Oxycodone Self-Administration

As per our previously published protocol, drug-naive rats were allowed to self-administer oxycodone-HCL (NIDA Drug Supply Program) (0.1 mg/kg/infusion) or saline, over 3.5 s (0.1 mL per infusion), in a sound-attenuated cabinet under an FR1 schedule with a 20 s timeout accompanied by a 5 s compound tone–light cue [20]. Briefly, rats (*n* = 36) were initially assigned to three groups: Saline (Sal) (*n* = 8), Short-access (*n* = 10), or Long-access (LgA) (*n* = 18). Short-access rats were trained to self-administer oxycodone for only one 3 h session for the entirety of the study (days 1–20). Long-access (LgA) and Sal rats were trained to self-administer for three sessions: one 3 h session during days 1–5, followed by two 3 h sessions during days 6–10, and then for three 3 h sessions during the rest of the study (days 11–20) (see Figure 1A). We gradually increased access to oxycodone over these weeks to prevent any adverse effects of oxycodone intake, including overdoses. This approach has been used successfully by us and other investigators [22,41]. There was a 20 s timeout between each infusion. Each 3 h session for LgA and Sal from day 6 to day 20 was separated by a 30 min timeout. This 30 min break was implemented to prevent overdoses, as there was no limit to the number of infusions a rat could take during a session. We also included a 48 h weekend abstinence period between every five days of SA to prevent overdose [25,26]. This schedule was implemented because this approach is effective at preventing significant weight loss that might have led to the elimination of some rats from the study; this approach also did not impact drug-taking behaviors [25,42,89,90,91,92]. Catheter patency was tested through the experiment. Rats were euthanized two hours after the first session of the last day. Saline animals underwent similar surgical procedures to oxycodone rats, were placed in the identical operant chambers, and experienced similar cue presentations during SA sessions, with saline being substituted for oxycodone.

### 4.4. RNA Extraction and Sequencing

Rats were euthanized two hours into their last self-administration session using rapid decapitation with a guillotine. Rat dorsal striata were visually dissected out using specific neuroanatomical coordinates (A/P +2 to −2 mm bregma, mediolateral ± 2 to 5 mm, dorsoventral −3 to −6 mm) and immediately snap-frozen on dry ice and stored at −80 °C [41]. Total RNA was extracted from the dorsal striatum using the Qiagen RNeasy Mini kit (Qiagen, Valencia, CA, USA), and RNA integrity (RIN) was checked using the Agilent bioanalyzer 2100 (Agilent, Santa Clara, CA, USA), and six samples per group with RIN 8 or above were shipped on dry ice to Azenta, Genewiz (Genewiz South Plainfield, NJ, USA) for RNA sequencing. RNA sequencing was selected as the first testing method as it is a commonly used method to quantify global transcriptional changes [35]. More details about the analysis of the RNA sequencing data are provided in the supplementary text, Section S1, and in the Results Section 2.2. The RNA-seq data has been deposited in NCBI under GEO accession number GSE280582.

### 4.5. Quantitative RT-PCR

Quantitative RT-PCR was performed as per our routine protocol [41]. Briefly, total RNA (0.5 μg) was reverse-transcribed to cDNA with oligo dT primers using Advantage RT-for-PCR kit (Takara Bio, San Jose, CA, USA). RT-qPCR was performed with Roche LightCycler 480 II (Roche Diagnostics, Indianapolis, IN, USA) using Luna Universal qPCR SYBR GREEN (NEB Inc., Ipswich, MA, USA) according to the manufacturer’s protocol. We purchased gene-specific primers from the Synthesis and Sequencing Facility of Johns Hopkins University (Baltimore, MD, USA). These primers were designed using Thermo Fisher Scientific, Waltham, MA, USA (OligoPrefect Primer Designer software version 6). Relative amounts of mRNA were normalized using beta-2 microglobulin (B2M) as a reference gene and reported as fold changes. The primer sequences used for PCR are listed in Supplementary Table S1. We selected these genes by looking for a pattern in the IPA and Sankey graphs made from genes with expression that was significantly different when compared to other groups in the RNA-seq data (Figure 3).

### 4.6. Statistical Analyses

Behavioral data was analyzed with the statistical program GraphPad Prism 10 using factorial ANOVA with repeated measures. LgA animals were assigned to different phenotypes based on our lab’s previously published articles [20,21,41,42,43,44]. A second-degree polynomial regression model was used to identify potential non-linear patterns in oxycodone intake over 20 days of SA for individual animals, to segregate the rats into LgA-H and LgA-L subgroups. The rats that escalated their intake were termed as LgA-H, while those who did not were termed as LgA-L. Biochemical data were analyzed using one-way ANOVA followed by Tukey’s multiple comparisons test if the main effect was significant. Linear regression analyses were performed to see if there were any correlations between gene expression and oxycodone intake. The slopes of all the regression lines were calculated using one-way ANOVA. Statistical significance for all hypothesis tests was set at *p* < 0.05.

## 5. Conclusions

In summary, LgA-H animals took higher quantities of oxycodone than LgA-L rats that were exposed to a similar schedule of long-access oxycodone self-administration. RNA sequencing analysis revealed the involvement of several gene networks, including some that participate in oxycodone-induced behaviors, in cognitive functions, and in opioid use disorder. Interestingly, the mRNA levels of several potassium channels were positively correlated to oxycodone intake, further implicating potassium in SUDs [34]. Finally, our observations underscore the importance of exploring the potential of potassium channel drugs in the treatment of OUD and other SUDs.

## Data Availability

The RNA sequencing data have been deposited at the NCBI GEO under the accession # GSE280582. All other data generated in this study will be made available upon reasonable request to the corresponding author via email.

## Supplementary Materials

The supporting information can be downloaded at: https://www.mdpi.com/article/10.3390/ijms26157356/s1.
