# Supplementary material for: Escalated Oxycodone Self-Administration Is Associated with Activation of Specific Gene Networks in the Rat Dorsal Striatum
Source: Int J Mol Sci. 2025 Jul 30;26(15):7356. doi: 10.3390/ijms26157356 (PMC12347817; doi:10.3390/ijms26157356)
Supplement: Supplementary file 1 [file ijms-26-07356-s001.zip › ijms-3748611-supplementary.pdf]

Supplementary File

Materials and Methods

1. **RNA extraction and sequencing**

Messenger RNA was extracted from 1ug of total RNA using the NEBNext Poly(A) mRNA Magnetic Isolation Module (New England Biolabs, Ipswich, MA, USA). RNA sequencing libraries were generated using the NEBNext Ultra II RNA Library Prep kit following manufacturer's instructions (New England Biolabs, Ipswich, MA, USA). Briefly, enriched mRNAs were fragmented for 15 minutes at 94 °C. First strand and second strand cDNAs were subsequently synthesized. cDNA fragments were end repaired and adenylated at 3'ends, and universal adapters were ligated to cDNA fragments, followed by index addition and library enrichment by limited-cycle PCR. The sequencing libraries were then validated on the Agilent TapeStation 4200 (Agilent Technologies, Palo Alto, CA, USA), and quantified by using Qubit 2.0 Fluorometer (Invitrogen, Carlsbad, CA) as well as by quantitative PCR (KAPA Biosystems, Wilmington, MA, USA). The sequencing libraries were clustered on two flowcell lanes. After clustering, the flowcells were loaded on the Illumina® HiSeq instrument (4000 or equivalent) according to manufacturer's instructions. The samples were sequenced using a 2x150bp Paired End (PE) configuration (GeneWiz, South Plainfield, NJ, USA). Image analysis and base calling were conducted by the HiSeq Control Software (HCS). Raw sequence data (.bcl files) generated from Illumina HiSeq was converted into fastq files and de-multiplexed using Illumina's bcl2fastq 2.17 software. One mismatch was allowed for index sequence identification.

After investigating the quality of the raw data, sequence reads were trimmed to remove possible adapter sequence and nucleotides with poor quality using Trimmomatic v.0.36. The trimmed reads were mapped to the Rattus norvegicus reference genome available on ENSEMBL using the STAR aligner v.2.5.2b. The STAR aligner is a splice aligner that detects splice junctions and incorporates them to help align the entire read sequences. BAM file were generated as a result of this step. Unique gene hit counts were calculated by using feature Counts from the Subread package v.1.5.2. Only unique reads that fell within exon regions were counted. After extraction of gene hi counts, the gene hit counts table was used for downstream differential expression analysis. Using DESeq2, a comparison of gene expression between the groups of samples was performed. The Wald test was used to generate p-values and Log2 fold changes. Genes with adjusted p values < 0.05 and absolute log2 fold changes > 1 were called as differentially expressed genes (DEGs) for each comparison. A gene ontology analysis was performed on the statistically significant set of genes by implementing the software Gene set clustering based on functional annotation. (GeneSCF).

Table S1: List of RT qPCR primer sequences

**Gene Name**	**Forward Primer**	**Reverse Primer**
Kcnma1	ATG AGA AGC CTG GAT GAC GTT T	TCA GCT TGG CTT GCT CTA TTG A
Kcnd3	CAG CCC CTA AAT CTC CAA CTG T	TCT CAG AAT TCT TCG GGC TCT G
Kcnk9	TTC TAT GCT GTG CTG GGT ATC C	TTC TTG ATC CGT TTC AGC AGG T
Kcng3	CTC TCC GCT GAG TTC CTG AAT T	CCC AGG GAG AAA CAC GTG AAT A
Kcnq1	GTG ATG TTG ACC ACT TCC GAA TAC	TCA CTT TAG GGG AGA AGT TGT CAG
Slc24a3	AAG CTA GCC AGT TTC ATC CCT C	TCT GAG GTG AAC CCA TTC TGT G
Cldn3	ACC TTC CAG ATG GTT ACA GAC G	TTCCTAGGCCAGGTAATCAGGT
Serping1	CAA GTT TCA GCC CAC TTA CGT G	CCC ACA CAG GTT GAG ATC GTA A
Serpingh1	GCC CAG ACA CTA TGA GAC CAA A	TGG CCA CGA ATA TGA ACA CTG A
Fmo2	CAG TGG GAC GTG TAT GTT CAG A	TAC CTG GAA ACG ACT TGA GTG G
Slc19a3	CTC ACT TTT CCT ACC CAT GCC T	CGT TGT TGT GAG CTT CCT TGA G
Nectin4	CCC TAC TGA CTG CTC CAC TTT T	ACA CAC GGA ATG ACC TAC ACT C
B2m (reference gene)	GAT CTT TCT GGT GCT TGT	AGC TCA ATT TCT ATT TGA GGT

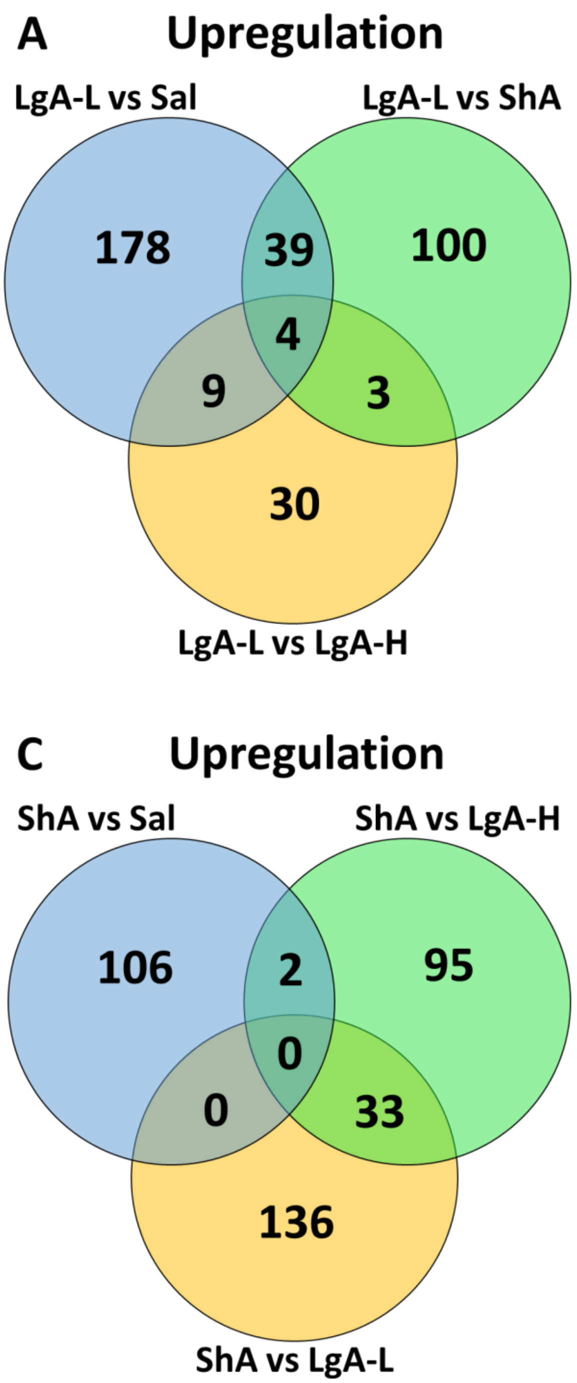

Supplementary Fig 1: Pairwise comparison of LgA-L with Sal, ShA and LgA-H; A) Upregulated genes, and B) Down-regulated genes. Pairwise comparison of Sha with Sal, LgA-L, and LgA-H; C) Upregulated genes, and D) Down-regulated genes

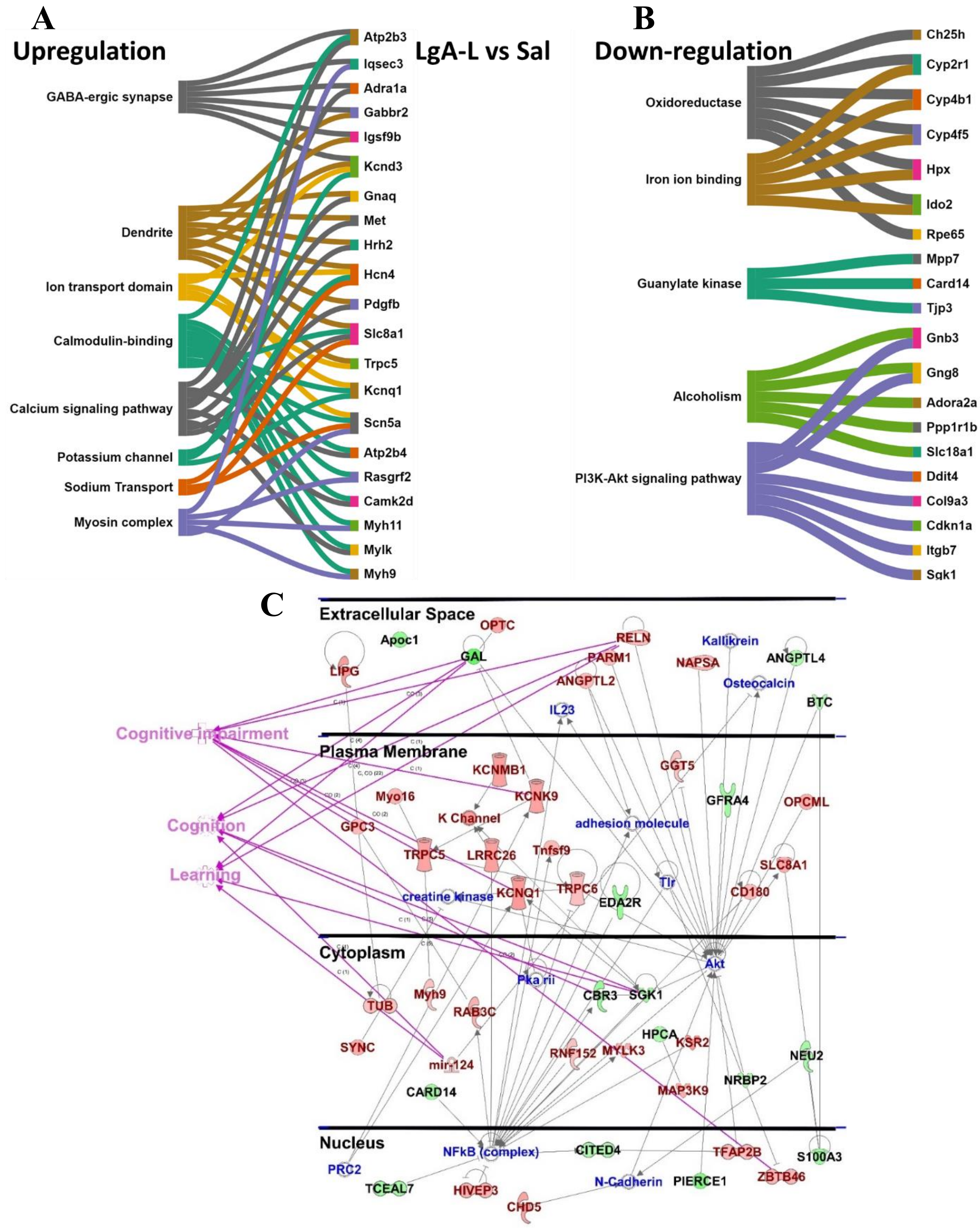

Supplementary Fig 2: DAVID annotation and Sankey diagrams revealed functional classification of DEGs A) Upregulated genes, and B) Down-regulated genes in LgA-L vs Sal comparison. C) Ingenuity Pathway Analysis shows the DEGS in LgA-L vs Sal comparison is involved in cognitive impairment, cognition, and learning. The red color represents upregulated genes whereas the green color represents downregulated genes.

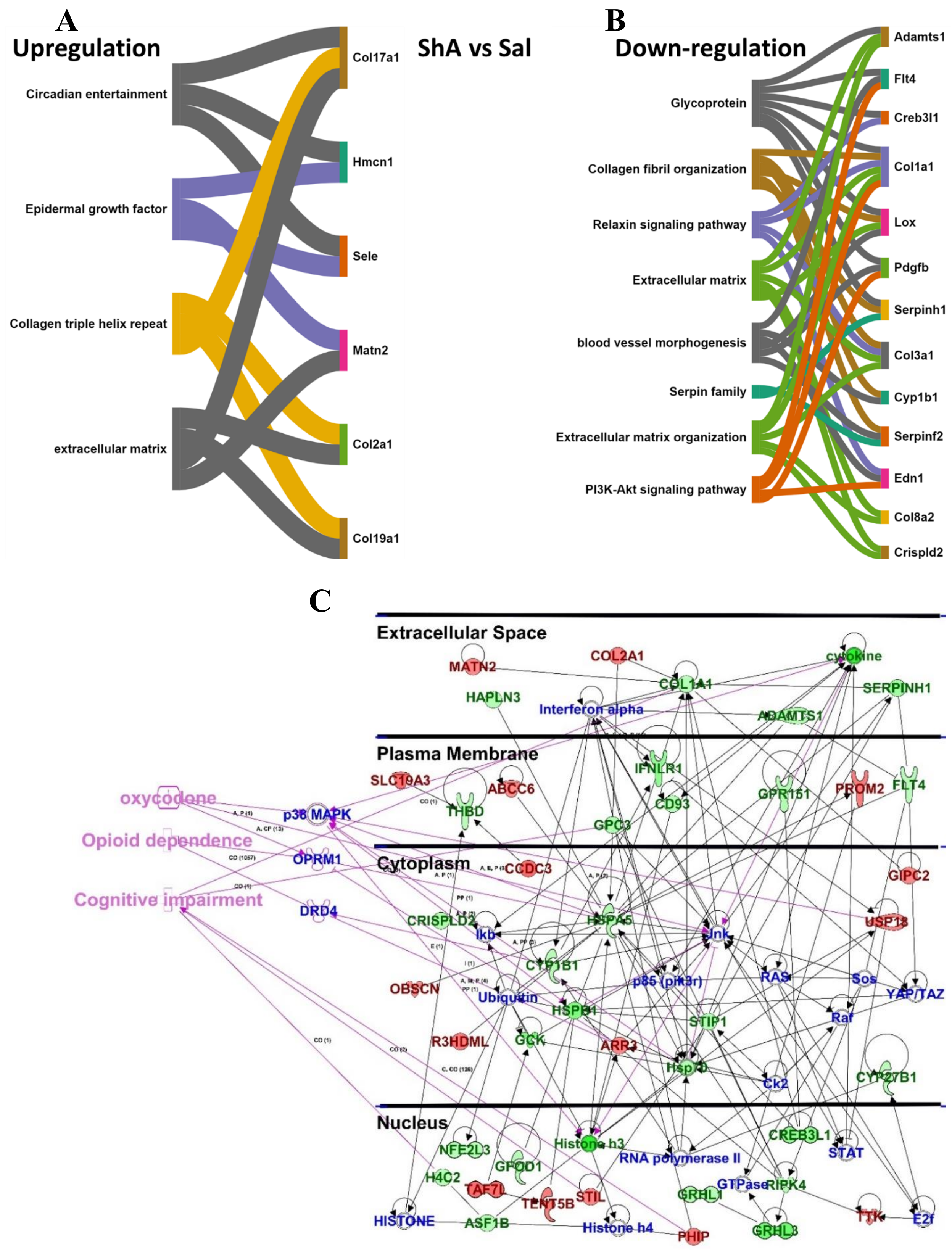

Supplementary Fig 3: DAVID annotation and Sankey diagrams revealed functional classification of DEGs A) Upregulated genes, and B) Down-regulated genes in ShA vs Sal comparison. C) Ingenuity Pathway Analysis shows the DEGS in ShA vs Sal comparison is involved in oxycodone intake, opioid dependence, and cognitive impairment. The red color represents upregulated genes whereas the green color represents downregulated genes.
